# Percentage of Myeloid Dendritic Cells in Peripheral Venous Blood Is Negatively Related to Incidence of Graves' Orbitopathy

**DOI:** 10.1155/2021/8896055

**Published:** 2021-01-26

**Authors:** Katarzyna Wojciechowska-Durczynska, Katarzyna Wieczorek-Szukala, Borys Stefanski, Arkadiusz Zygmunt, Jan Stepniak, Małgorzata Karbownik-Lewinska, Andrzej Lewinski

**Affiliations:** ^1^Department of Endocrinology and Metabolic Disease, Medical University of Lodz, 93-338 Lodz, Poland; ^2^Polish Mother's Memorial Hospital-Research Institute, 93-338 Lodz, Poland; ^3^Department of Oncological Endocrinology, Medical University of Lodz, 90-752 Lodz, Poland

## Abstract

The aim of the study was to evaluate the distribution of blood dendritic cells (DCs) in patients with Graves' orbitopathy (GO) and to assess the influence of methylprednisolone therapy on subsets of peripheral blood mononuclear cells (PBMCs). Peripheral blood DC subsets were analyzed by flow cytometry in patients with active GO (*n* = 17), inactive GO (*n* = 8), and Graves' disease (GD) without GO (*n* = 8) and controls (*n* = 15); additionally, in patients with active GO (*n* = 17), analyses were done at three time points, i.e., before methylprednisolone treatment and after 6 weeks and after 12 weeks of the treatment. Percentage of myeloid DCs (mDCs) in PBMC fraction was significantly lower in patients with both active and inactive GO, compared to patients with GD without GO and controls (*p* < 0.05). In addition, mDCs were also documented to be an independent factor negatively associated with GO, however without essential differences between active and inactive phases. On the other hand, we did not observe any changes in the percentage of DCs after methylprednisolone therapy (*p* > 0.05). In the present study, we have succeeded to firstly demonstrate—according to our knowledge—that blood mDCs are negatively related to GO incidence.

## 1. Introduction

Graves' orbitopathy (GO) is the most common extrathyroidal manifestation of Graves' disease (GD). It has a self-limited active phase, followed by an inactive phase. Graves' orbitopathy remains a difficult clinical problem, and treatment options of severe active disease have been limited to steroids in the majority of cases [1]. The lack of development of specific medical therapies for GO results—in large part—from poor understanding of disease pathogenesis. While major breakthroughs continue to occur in closely related thyroid autoimmune diseases, a progress in identifying the pathogenic mechanisms relevant to GO is not satisfactory.

Regulation of immunity involves very complex systemic and local processes, depending on the activity of many regulatory cell types. The breakdown of central and peripheral immune tolerance to autoantigens in autoimmune diseases, including GO, is largely due to deficient immune regulation. The mechanism preventing autoagressive processes includes immune cell populations involved in central and peripheral tolerance—mainly dendritic cells (DCs).

Dendritic cells are capable of either triggering autoreactive T cells or activating regulatory T cells, depending on the maturation stimuli and/or DC subset. Additionally, as the most effective antigen-presenting cells (APCs), DCs are important regulators of the adoptive immunity, linking in that way both major arms of the host defence.

Circulating human DCs, which typically constitute 0.5-2% of peripheral blood mononuclear cells (PBMCs), are classified as myeloid DCs and plasmacytoid DCs [2]. They differ in phenotype and function. The myeloid subset is thought to be responsible for the induction and propagation of Th1- and Th17-driven immune responses [3]. The plasmacytoid DCs are characterized by the ability to secrete large amounts of type I interferon and induce differentiation of Th2 cells [4], as well as of regulatory cells [5, 6].

Other important PBMCs are monocytes. Monocytes act also as APCs and have been categorized into a subset based on differential expression levels of CD14 and CD16. On the basis of these parameters, monocytes were divided into the main “classic” fraction, characterized by very high expression of CD14, and a much smaller subpopulation—characterized by high surface expression of CD16 [7, 8]. The factors that influence the distribution of monocyte subsets and the role that each subset plays in autoimmunity are not well documented.

The aim of the study was to determine whether there are any differences concerning DCs between patients with active and/or inactive GO and patients with GD without GO or healthy controls and in what way methylprednisolone treatment affects the distribution of the DC subpopulation. To our knowledge, this study is the first attempt assessing subsets of blood DCs, carried out in GO patients regarding DC activity and the effects exerted by immunosuppressive therapy.

## 2. Materials and Methods

The study participants have been recruited from the Department of Endocrinology and Metabolic Diseases, Medical University of Lodz. Prior to enrollment, all participants signed the informed consent, according to the study protocol approved by the Ethics Committee of the Medical University of Lodz (RNN/206/12/KE), and filled in a GO quality-of-life questionnaire. The study has included 17 patients with active GO of severity from moderate to severe, 8 patients with inactive mild GO, 8 patients with GD without GO, and 15 healthy euthyroid volunteers age- and sex-matched to the research group (controls). Complete clinical and demographic data of all subjects were registered (Table 1).

All GO patients, without other eye diseases or orbital tumors, and GD patients without GO have been finally diagnosed by the presence of clinical manifestations and by laboratory examinations. To assess GO activity, we have utilized the clinical activity score (CAS) and magnetic resonance imaging (MRI). The control subjects were euthyroid and had no autoimmune diseases, including autoimmune thyroid diseases. All patients with active GO have been treated with intravenous methylprednisolone. Therapy schedule—based on EUGOGO recommendations—comprised 12 weekly infusions (6 weekly infusions of 0.5 g, followed by 6 weekly infusions of 0.25 g).

Peripheral blood samples have been collected after an overnight fast. Venous blood has been obtained by clean venipuncture (needle gauge 19), avoiding slow flowing draws and/or traumatic venipunctures. The blood samples have been collected from the same patient with active GO at three consecutive time points: directly before the administration of steroids and after 6 weeks and after 12 weeks of steroid course. The blood samples taken before steroid administration from active GO patients were used for further comparative analysis among four groups.

### 2.1. Fluorescence-Activated Cell Sorting (FACS) Analysis

Whole blood samples obtained from study participants have been assessed on the same day by flow cytometry, using a FACSCanto II® cytometer and FACSDiva® software (BD Biosciences, San Jose, CA, USA).

Peripheral blood DC subsets have been recognized on the basis of the surface expression pattern of blood dendritic cell antigens (BDCAs). The staining of peripheral blood DCs has been performed as follows:

- CD141/BDCA3 (AD5-14H12, mouse IgG1) for mDCs
- CD303/BDCA2 (AC144, mouse IgG1) for pDCs

Monoclonal antibodies (mAb) specific for BDCA antigens were purchased from Miltenyi Biotec (Bergisch Gladbach, Germany). All remaining mAb and appropriate isotype controls were purchased from BD Biosciences Pharmingen (San Jose, CA, USA).

Other cell populations were stained with the following antibodies:

- Positive for CD16: anti-CD16 antibody (3G8, mouse IgG1)
- Positive for CD19: anti-CD19 antibody (4G7, mouse IgG1)
- Positive for CD14: anti-CD14 antibody (M5E2, mouse IgG2a)
- Positive for CD3: anti-CD3 antibody (UCHT1, mouse IgG1)

After the incubation with mAb, erythrocytes have been lysed (15 minutes at room temperature) with FACS Lysing Solution (BD Biosciences, San Jose, CA, USA). The leukocyte fraction then has been washed twice with cold phosphate-buffered saline (PBS), counted, and suspended in PBS for FACS analysis. To avoid an unspecific antibody binding, an Fcr-blocking reagent (Miltenyi Biotec, Bergisch Gladbach, Germany) has been applied in all analyses. The DC subpopulation has been analyzed in a blinded way, and the results have been expressed as a percentage of PBMC fraction.

**Figure 1:**
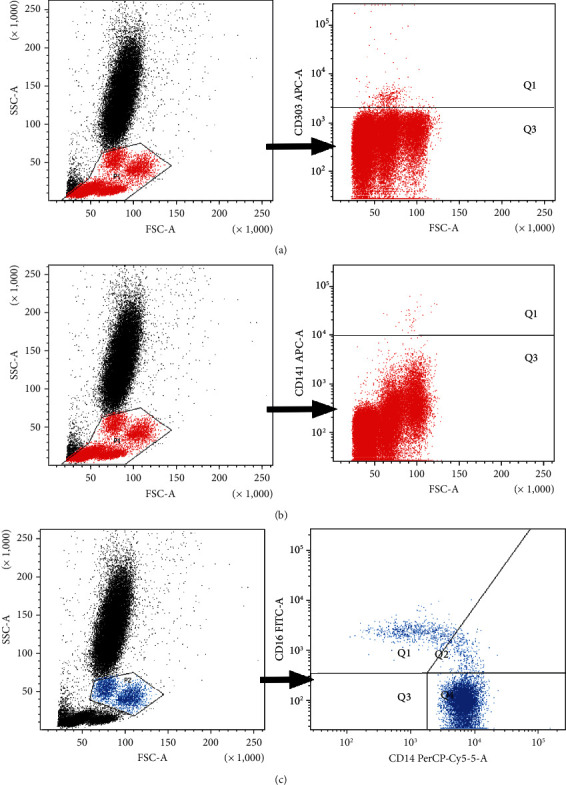
Exemplary plots of flow cytometry analysis showing the gating strategy of analyzed leukocyte populations. (a) Distribution of cellular populations in forward and side scatter (FSC-SSC) picture, PBMCs gated for further DC analysis (gate P1), and exemplary plots of flow cytometry analysis showing the staining with antibodies specific for pDCs in mononuclear leukocyte fraction: BDCA2/CD303 pDCs (quadrant Q1). (b) Distribution of cellular populations in forward and side scatter (FSC-SSC) picture, PBMCs gated for further DC analysis (gate P1), and exemplary plots of flow cytometry analysis showing the staining with antibodies specific for mDCs in mononuclear leukocyte fraction: BDCA3/CD141 mDCs (quadrant Q1). (c) Distribution of cellular populations in forward and side scatter (FSC-SSC) picture, monocytes gated for further analysis (gate P2, blue population), and exemplary plots of flow cytometry analysis showing the staining with antibodies specific for CD14 and CD16 in monocyte fraction. Gating of individual monocyte subpopulations is presented: monocytes (CD14highCD16−) (quadrant Q4), monocytes (CD14highCD16+) (quadrant Q2), and monocytes (CD14lowCD16+) (quadrant Q1).

Exemplary plots of flow cytometry analysis showing the gating strategy of analyzed leukocyte populations are shown in Figure 1.

### 2.2. Statistical Analysis

Statistical analyses were performed using Statistica 13.1. The ANOVA Kruskal-Wallis test was used to determine the significance of differences in all parameters among patients with active GO, inactive GO, and GD without GO and healthy controls. The ANOVA Friedman test and Wilcoxon matched-pair test were used to determine the significance of differences in all measured parameters among patients before, during, and after methylprednisolone therapy. Univariate and multivariate logistic regression analyses were used to determine which continuous variable might have been associated with GO [1]. Further analyses concerning correlations between TSH, thyroid hormones, TSH receptor antibodies, and thyroid treatment (thyrostatics, ^131^I) with immune elements were performed by the Spearman rank-order correlation. A *p* value less than 0.05 was considered statistically significant.

## 3. Results

**Figure 2:**
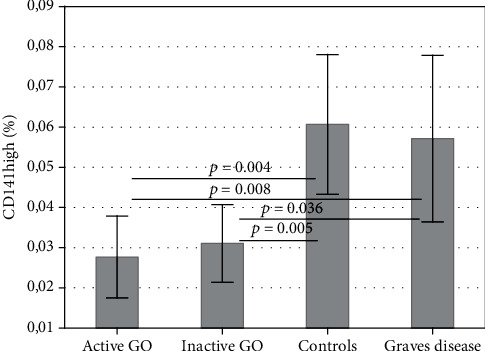
Percentage of mDCs (CD141+) in the peripheral blood of patients with active and inactive GO and controls and GD patients without GO. Data are presented as means ± standard deviation (SD) (ANOVA Kruskal-Wallis test).

The quantitative flow cytometric analysis showed that the percentage of mDCs (CD141) in whole PBMC fraction is significantly lower in GO (active and inactive) in comparison to GD without GO and controls, without differences observed between the latter two groups (*p* < 0.05) (Figure 2).

**Figure 3:**
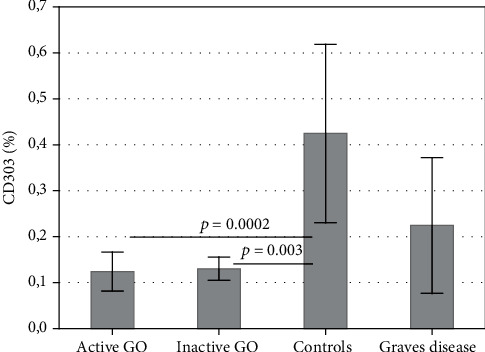
Percentage of pDCs (CD303+) in the peripheral blood of patients with active and inactive GO and controls and GD patients without GO. Data are presented as means ± standard deviation (SD) (ANOVA Kruskal-Wallis test).

The percentage of pDCs (CD303) is significantly lower in GO (active and inactive) when compared to controls (*p* < 0.05), but it is not proven to be lower in comparison to GD patients without GO (*p* > 0.05); at the same time, however, no statistical differences were found between GD patients without GO and controls (Figure 3).

**Figure 4:**
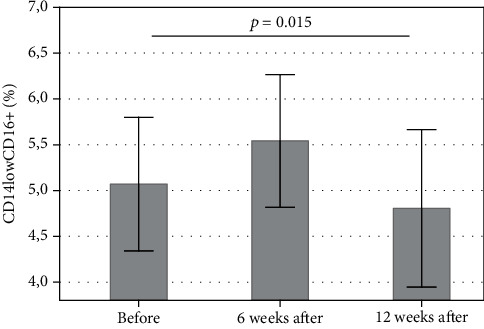
Percentage of CD14lowCD16+ at three (3) consecutive time points: before methylprednisolone treatment and after 6 weeks and after 12 weeks of the treatment. Statistical significance was found between the 1^st^ time point (i.e., before the treatment) and the last time point (after 12 weeks of treatment) (*p* = 0.015), as evaluated by the ANOVA Friedman test and Wilcoxon matched-pair test. Data are presented as means ± standard deviation (SD).

The administration of methylprednisolone significantly decreased the distribution of CD14lowCD16+ (%) after 12 weeks of treatment, compared with the time point before treatment (*p* < 0.05) (Figure 4). As regards the percentage of DCs, the administration of methylprednisolone did not influence the distribution of pDCs (*p* > 0.05) and mDCs (*p* > 0.05).

Subsequent analyses of correlations between clinical parameters (TSH, thyroid hormones, TSHR antibody levels, and thyroid treatment) with the immune cell populations are presented in Table 2. The population of pDCs showed a negative correlation with thyrostatic treatment, and monocytes proved to be positively correlated with free thyroid hormone levels and ^131^I therapy (Table 2). No correlations were found between mDCs and all variables considered (Table 2).

For the group of GO, variables such as TSH, FT3, FT4, and TSHR antibody levels as well as linear variables concerning populations of immune cells were subjected to a univariate regression model. The purpose of this model was to determine which of those continuous variables might have been associated with GO. pDCs (CD303) and mDCs (CD141) did constitute linear factors negatively associated with GO, whereas TSHR antibodies are positively related to GO (Table 3).

Therefore, these three variables, i.e. pDCs, mDCs, and TSHR antibodies, were subjected to the second step of regression analysis, that is, multivariate regression analysis, and mDCs (CD141) were proven to be the only independent factor negatively associated with GO (Table 4). Our results analyzed by using a logistic regression model are very similar to those obtained by the ANOVA Kruskal-Wallis test. Although the bigger sample is usually recommended in statistics, statisticians generally accept to perform the regression analysis in case of much lower sample size and in case of special circumstances [1], especially when the results can be expected or when they are confirmed by other statistical tests.

Finally, for the group of GO (active plus inactive), linear variables concerning populations of immune cells were submitted to a univariate regression model. The purpose of this model was to determine which of those continuous variables might be independently associated with active GO; however, all measured variables proved to be not related (data not shown).

## 4. Discussion

Graves' disease is a complex autoimmune disease, in which not only the thyroid gland but also the orbit tissues are affected. Therefore, we decided to evaluate the distribution of DCs in the peripheral blood of patients with GO in the course of GD, considering the complexity of this pathogenesis.

So far, several studies reported the presence of DCs in GD. In the study by Leskela and coworkers, the authors demonstrated reduced number of pDCs in peripheral circulation in patients with severe GD; concomitantly, the number of pDCs in the thyroid gland was increased [9]. Similar results were obtained by Hassan and coworkers; additionally, there were a greater number of pDCs in the neck lymph nodes of patients with GD, compared to patients with nodular goitre [10]. It suggests that there is a migration of pDCs from peripheral blood and accumulation in the thyroid and the neck lymph nodes in patients with GD. In contrast, Mao and coworkers reported the highest proportion of pDCs in the case of untreated GD (uGD), compared to the control group, and the highest ratio of pDC/DC in uGD compared to controls and euthyroid patients with autoimmune disease (GD and Hashimoto thyroiditis) [11]. Thus, there is some discrepancy in the literature regarding the distribution of DCs in GD. In our study, we have observed a reduced number of pDCs in GD compared to healthy controls (Figure 2); however, this difference did not reach statistical significance, possibly due to nonhomogenous distribution of pDC numbers. In addition, the presence of DC subsets in GO has not been characterized till now.

Results of our study confirmed diminished percentage of mDCs in peripheral blood in patients with active and inactive GO in comparison to healthy controls and GD patients without GO. Of great importance is the observation that among numerous linear variables (including TSHR antibodies), one type of mDCs turned out to be an independent factor associated (negatively) with GO. Thus, on the basis of our findings, it can be concluded that mDCs are more strongly associated with GO than TSHR antibodies are.

In previous studies, it has been shown that thyrometabolic status can influence the subsets of dendritic cells. The results of the study conducted by Dedecjus and coworkers indicated that L-thyroxine replacement therapy during a hypothyroid state would increase mDCs and pDCs [12]. In the present study, we have confirmed no relationship between mDCs and thyroid tests (TSH, FT3, FT4, and TSHR antibodies), which suggests that the presence of GO may be a factor more strongly affecting the distribution of these types of DCs than thyroid dysfunction. Of importance is also the finding that neither the treatment with thyrostatics nor ^131^I therapy did affect mDC level. Instead, the results of our study confirmed that the pDC percentage is negatively correlated with thyrostatic therapy, which partially explains prior results of the study by Mao and coworkers that demonstrated the highest proportion of pDCs in the case of uGD [11]. However, the significance of pDCs in relation to GO pathogenesis requires further studies.

Concerning mDCs, we documented that blood level of this type of DCs does not depend on the activity of GO. Thus, on the basis of our results, it can be stated that mDCs are strongly associated just with the presence of GO without the contribution of other clinical or biochemical factors.

Myeloid dendritic cells are a very small population of immune cells, comprising only hundredth of a percent of PBMCs in healthy individuals. Although the data is limited, a decrease in mDCs might have a discriminative role in GO pathogenesis. The diminution of mDCs in GO patients could be this divergence between GD and GO pathogenesis that explains why orbitopathy occurs in only some cases of GD.

Furthermore, our results generated the hypothesis that mDCs might migrate to the orbit tissues in GO. The study by Eckstein and coworkers, which characterized the periorbital immune cells in active and inactive thyroid-associated ophthalmopathy, demonstrated that DCs were absent in orbit tissues in all examined conditions [13]. Nevertheless, the results of the study by Fang and coworkers confirmed that GO orbital microenvironment was composed of dendritic cells. Further analysis demonstrated that GO orbital connective tissues expressed genes representing antigen-presenting cells, and most interestingly, control orbital tissues have much fewer antigen-presenting cells in comparison to those of GO [14]. Moreover, the results of the aforementioned study confirmed the involvement of Th17 cell pathway response in GO [14]. As previously mentioned, the mDCs are associated with the Th17 response; thus, taking together the results of our study and the study by Fang and coworkers, the migration of mDCs from circulation to the orbital tissues and their participation in GO can be expected. Therefore, in our opinion, the additional studies should be performed to clarify this interesting aspect. On the basis of our results, the migration of pDCs to the orbital tissue cannot be excluded; however, our results do not speak in favour of such a possibility.

Based on the above-mentioned observation, we considered the possibility that not only thyrometabolic status might correlate with the number of DCs but also the immunosuppressive treatment. To investigate this possibility, we conducted investigation in patients with active GO treated with methylprednisolone.

In our study, the administration of methylprednisolone has not influenced the percentage of DC subsets. The results we have obtained confirm our previous findings that GO activity and change of this activity by steroids do not affect DC populations.

On the other hand, we confirmed the decrease in the population of CD14lowCD16+ monocytes after methylprednisolone treatment. In concordance with our findings, the aforementioned study by Eckstein and coworkers confirmed decrease in the infiltration of periorbital tissues by monocytes in GO after steroid treatment. Therefore, alteration of monocytes may play a relevant role in the immunosuppressive activity of steroids in GO. Our findings may constitute the basis for future studies, aimed at investigating the association between response to immunosuppressive treatment and immunological status in GO. Some limitation of our results is a relatively small number of GO patients that somehow weakens the final conclusions and makes that they should be considered with caution.

## 5. Conclusions

To the best of our knowledge, no reports related to the role of DCs in GO have been published before. In our present study, we have succeeded to demonstrate that blood mDCs are negatively related to GO incidence. The change of the mDC proportions could have a contribution to GO pathogenesis. It is to be recalled that one of 4 groups (patients with active GO) was assessed in the presence of methylprednisolone treatment; however, that steroid therapy had not influenced the percentage of DC subsets.

Certainly, the elucidation of autoimmune processes in GO may lead to the development of novel therapeutic strategies, regarding immunocompetent cells like DCs.

## Figures and Tables

**Table 1:** Characteristics of patients and controls included in the study.

Characteristics	Active GO patients (*n* = 17)	Inactive GO patients (*n* = 8)	Controls (*n* = 15)	GD without GO (*n* = 8)
Age at study enrollment, mean ± SD	54.53 ± 10.86	50 ± 16.37	43.73 ± 19.713	53.13 ± 16.35
Gender				
Female	14	7	12	6
Male	3	1	3	2
TSH (mIU/L)	1.10 ± 1.52	1.29 ± 1.6	1.32 ± 0.42	0.49 ± 1.34
Free T3 (pg/mL)	4.52 ± 3.86	5.63 ± 7.81	3.01 ± 0.55	7.12 ± 4.66
Free T4 (ng/dL)	1.29 ± 0.76	1.73 ± 1.74	1.26 ± 0.14	2.12.±1.31
Euthyroid^a^				
Yes	12	5	15	1
No	5	3	0	7
TR-Ab^b^ (IU/L)				
Positive	17	6	0	8
Negative	0	2	9	0
Unknown	0	0	6	0
TPO-Ab^c^ (IU/mL)				
Positive	8	4	0	6
Negative	9	3	9	0
Unknown	0	1	6	1
Tg-Ab^d^ (IU/mL)				
Positive	5	4	0	3
Negative	12	3	9	3
Unknown	0	1	6	2
CAS				
>3	17	0		
<3	0	8		
Antithyroid treatment				
Yes	15	3		7
No	2	5		1
L-Thyroxine				
Yes	1	2		0
No	16	6		8
^131^I				
Yes	2	0		0
No	15	8		8
Surgery				
Yes	1	1		0
No	16	7		8
Smoking				
Yes	14	3		1
No	0	5		0
Unknown	3	0		7
MRI				
Active	12	0		
Inactive	0	6		
Unknown	5	2		

^a^Patients were considered euthyroid when TSH is 0.27–4.2 mIU/L, FT3 is 2.6-4.4 pg/mL, and FT4 is 0.93–1.7 ng/dL. ^b^TR-Ab TSH receptor antibody, negative <1.75 IU/L. ^c^TPO-Ab thyroid peroxidase antibody, negative <34 IU/mL. ^d^Tg-Ab thyroglobulin antibody, negative <115 IU/mL.

**Table 2:** The results of the Spearman rank-order correlation analysis. The pairs of variables with positive correlation coefficients and *p* values below 0.050 tend to increase together. For the pair with negative correlation coefficients and *p* values below 0.050, one variable tends to decrease while the other increases. For pairs with *p* values greater than 0.050, there is no significant relation between the two variables.

	TSH	FT3	FT4	THSR antibodies	Thyrostatics	^131^I
CD14^+^	*p* = 0.296	*p* = 0.686	*p* = 0.528	*p* = 0.952	*p* = 0.10	*p* = 0.00
CD16^+^	*p* = 0.968	+*p* = 0.0526	+*p* = 0.0111	*p* = 0.916	*p* = 0.25	+*p* = 0.03
CD14^−^CD16+	*p* = 0.557	*p* = 0.192	*p* = 0.101	*p* = 0.914	*p* = 0.178	*p* = 0.055
CD14highCD16-	*p* = 0.272	*p* = 0.548	*p* = 0.425	*p* = 0.984	*p* = 0.102	*p* = 0.07
CD14^+^CD16^+^	*p* = 0.306	*p* = 0.103	+*p* = 0.0266	*p* = 0.746	*p* = 0.28	*p* = 0.09
CD14highCD16-	*p* = 0.763	*p* = 0.485	*p* = 0.104	*p* = 0.965	*p* = 0.307	*p* = 0.187
CD14lowCD16+	*p* = 0.943	*p* = 0.526	*p* = 0.276	*p* = 0.943	*p* = 0.255	*p* = 0.07
CD14highCD16+	*p* = 0.454	*p* = 0.242	*p* = 0.0510	*p* = 0.629	*p* = 0.306	*p* = 0.149
CD3^+^	*p* = 0.135	*p* = 0.969	*p* = 0.835	*p* = 0.507	*p* = 0.051	*p* = 0.149
CD19^+^	*p* = 0.559	*p* = 0.202	*p* = 0.362	*p* = 0.133	*p* = 0.051	*p* = 0.149
CD303^+^	*p* = 0.754	*p* = 0.753	*p* = 0.188	*p* = 0.644	−*p* = 0.028	*p* = 0.245
CD141^+^	*p* = 0.517	*p* = 0.717	*p* = 0.871	*p* = 0.446	*p* = 0.19	*p* = 0.204

**Table 3:** Univariate logistic regression analysis of the univariate GO determinants (variables), performed in all patients, i.e., with and without (controls, GD) GO. OR: odds ratio; CI: confidence interval. Values *p* < 0.05 are considered statistically significant.

Variable	Univariate regression		
	OR	95% CI	*p*
TSH	1.08	0.68-1.74	*p* = 0.718
FT4	1.01	0.88-1.16	*p* = 0.819
FT3	0.82	0.45-1.49	*p* = 0.505
TSHR antibodies	1.11	1.01-1.22	*p* = 0.022
CD14+	0.95	0.89-1.02	*p* = 0.133
CD16+	0.95	0.86-1.04	*p* = 0.248
CD14-CD16+	0.94	0.84-1.05	*p* = 0.239
CD14highCD16-	0.94	0.87-1.02	*p* = 0.127
CD14+CD16+	0.92	0.64-1.31	*p* = 0.643
CD3+	1.03	0.98-1.08	*p* = 0.193
CD14highCD16-	1.00	0.90-1.11	*p* = 0.970
CD14lowCD16+	0.94	0.78-1.13	*p* = 0.513
CD14highCD16+	1.13	0.92-1.39	*p* = 0.22
CD19+	1.11	0.98-1.27	*p* = 0.09
CD303+	0.0000046	1 × 10^−9^-0.02	*p* = 0.003
CD141+	5.4 × 10^−32^	0-1.3 × 10^−9^	*p* = 0.002

**Table 4:** Multivariate logistic regression analysis of the multivariate GO determinants (variables), performed in all patients, i.e., with and without (controls, GD) GO. OR: odds ratio; CI: confidence interval. Values *p* < 0.05 are considered statistically significant.

Variable	Multivariate regression		
	OR	95% CI	*p*
CD303+	0.000045	4 × 10^−9^-4.504	0.078
CD141+	0.000000	0-1.5 × 10^−8^	0.010
TSHR antibodies	1.11	0.98-1.25	0.073

## Data Availability

The data from flow cytometry analysis and statistical analysis used to support the findings of this study are available from the corresponding author upon request.
